# 12‐hr shifts in nursing: Do they remove unproductive time and information loss or do they reduce education and discussion opportunities for nurses? A cross‐sectional study in 12 European countries

**DOI:** 10.1111/jocn.14977

**Published:** 2019-10-28

**Authors:** Chiara Dall’Ora, Peter Griffiths, Talia Emmanuel, Anne Marie Rafferty, Sean Ewings, the RN4CAST Consortium, Walter Sermeus, Koen Van den Heede, Luk Bruyneel, Emmanuel Lesaffre, Linda Aiken, Herbert Smith, Douglas Sloane, Anne Marie Rafferty, Simon Jones, Jane Ball, Juha Kinnunen, Anneli Ensio, Virpi Jylhä, Reinhard Busse, Britta Zander, Miriam Blümel, John Mantas, Marianna Diomidous, Anne Scott, Anne Matthews, Anthony Staines, Ingeborg Strømseng Sjetne, Inger Margrethe Holter, Tomasz Brzostek, Maria Kózka, Piotr Brzyski, Teresa Moreno‐Casbas, Carmen Fuentelsaz‐Gallego, Esther Gonzalez‐María, Teresa Gomez‐Garcia, Carol Tishelman, Rikard Lindqvist, Lisa Smeds‐Alenius, Sabina De Geest, Maria Schubert, René Schwendimann, Dietmar Ausserhofer, Theo van Achterberg, Maud Heinen, Lisette Schoonhoven

**Affiliations:** ^1^ School of Health Sciences University of Southampton Southampton UK; ^2^ National Institute for Health Research Collaboration for Leadership in Applied Health Research and Care (NIHR CLAHRC) Wessex Southampton UK; ^3^ Department of Learning, Informatics, Management and Ethics, Division of Innovative Care Research Karolinska Institutet Stockholm Sweden; ^4^ Florence Nightingale Faculty of Nursing, Midwifery & Palliative Care King's College London London UK

**Keywords:** 12‐hr shifts, communication, continuity of patient care, education, continuing, nursing, patient handoff, shift work schedule

## Abstract

**Aims and objectives:**

To examine the association between registered nurses' (referred to as “nurses” for brevity) shifts of 12 hr or more and presence of continuing educational programmes; ability to discuss patient care with other nurses; assignments that foster continuity of care; and patient care information being lost during handovers.

**Background:**

The introduction of long shifts (i.e., shifts of 12 hr or more) remains controversial. While there are claims of efficiency, studies have shown long shifts to be associated with adverse effects on quality of care. Efficiency claims are predicated on the assumption that long shifts reduce overlaps between shifts; these overlaps are believed to be unproductive and dangerous. However, there are potentially valuable educational and communication activities that occur during these overlaps.

**Design:**

Cross‐sectional survey of 31,627 nurses within 487 hospitals in 12 European countries.

**Methods:**

The associations were measured through generalised linear mixed models. The study methods were compliant with the STROBE checklist.

**Results:**

When nurses worked shifts of 12 hr or more, they were less likely to report having continuing educational programmes; and time to discuss patient care with other nurses, compared to nurses working 8 hr or less. Nurses working shifts of 12 hr or more were less likely to report assignments that foster continuity of care, albeit the association was not significant. Similarly, working long shifts was associated with reports of patient care information being lost during handovers, although association was not significant.

**Conclusion:**

Working shifts of 12 hr or more is associated with reduced educational activities and fewer opportunities to discuss patient care, with potential negative consequences for safe and effective care.

**Relevance to clinical practice:**

Implementation of long shifts should be questioned, as reduced opportunity to discuss care or participate in educational activities may jeopardise the quality and safety of care for patients.

**What does this paper contribute to the wider global clinical community?**

- Reducing handovers through the introduction of shifts of 12 hr or more is not associated with enhanced continuity of patient care or reduced loss of patient information.
- Shorter 8‐hr shifts, which typically offer an extended overlap between early and late shifts, are associated with higher likelihood for nurses to have continuing educational programmes and to discuss patient care with colleagues.
- The assumption that implementing a two (shifts of 12 hr or more) shift system removes unproductive time and decreases risk of patient information loss is unwarranted. Shifts of 12 hr or more shifts appear to introduce a series of unintended consequences in terms of quality of patient communication.

## 1 INTRODUCTION

Shifts of 12 hr or more, also referred to as “long shifts” for hospital nursing have been introduced in many countries (Griffiths et al., 2014). These long shifts offer the opportunity to remove one handover between shifts each day and to reduce overlaps between shifts, leading to fewer staffing hours to be paid for (Ganong, Ganong, & Harrison, 1976; NHS Evidence, 2010). Efficiency savings from long shifts are predicated on the assumption that the reduction in overlaps removes unproductive time which does not “add value” to the delivery of care. Indeed, it has been claimed that reducing the number of handovers—thereby removing unproductive time—may have additional benefits including reducing associated risks and increasing continuity of care (NHS Evidence, 2010). Handovers are high‐risk processes that can lead to inconsistency in continuity of patient care and, as a result, to patient safety incidents. This is mainly due to miscommunication between healthcare providers (Cohen & Hilligoss, 2010; Raduma‐Tomas, Flin, Yule, & Williams, 2011).

## 2 BACKGROUND

Amongst the predicated benefits of the removed handovers resulting from shifts of 12 hr or more, there is increased patient safety due to reduced information loss. Since incidents often occur when responsibility is transferred from one person to another, reducing the number of transfers in a 24‐hr period decreases the opportunity for information to be lost or miscommunicated (Baillie & Thomas, 2019). An additional claimed benefit of long shifts and the associated reduction in handovers is that patients interact with the same nurse all day; there are reports that patients become confused when their carer changes during the day, hence the claim that shifts of 12 hr or more can improve continuity of care (Haller, Quatrara, Letzkus, & Keim‐Malpass, 2018; NHS Evidence, 2010; Thomson, Schneider, & Hare Duke, 2017; Wootten, 2000).

While the reduction in the number of handovers and overlaps of staff between shifts may lead to fewer opportunities for miscommunication, the overlap, sometimes up to 2 hr, arguably provides more opportunity to discuss patient care. The extended overlap between shifts is also a time that nurses have traditionally used to access both formal and informal education opportunities (Baillie & Thomas, 2019). Some early small scale reports suggested that nurses moving to shifts of 12 hr or more had fewer opportunities to participate in continuing education programmes compared to nurses working 8‐hr shifts (McGettrick & O'Neill, 2006; Reid, Todd, & Robinson, 1991).

Furthermore, there is a growing number of studies suggesting that shifts of 12 hr or more are associated with adverse outcomes for both patients and nurses, with nurses reporting both lower quality of care and increased omissions in necessary care to be associated with working long shifts (Ball et al., 2017; Dall’Ora, Griffiths, et al., 2019; Dall'Ora, Ball, Recio‐Saucedo, & Griffiths, 2016; Griffiths et al., 2014; Stimpfel & Aiken, 2013). These findings raise the possibility that far from reducing unproductive time, which adds little value to nursing care, the move to shifts of 12 hr or more may negatively impact nurses' ability to deliver safe and effective care.

The potential benefits of reduced number of handovers resulting from implementing shifts of 12 hr or more have never been formally tested; therefore, in this study, we aimed to examine the association between nurses' shifts of 12 hr or more and time for active staff development or continuing education activities; opportunity to discuss patient care with other nurses; assignments that foster continuity of care; and important patient information being lost during handover.

## 3 METHODS

This was a cross‐sectional survey study using nurse‐reported data from a large European survey, the RN4CAST study, which was conducted in 12 countries: Belgium, England, Switzerland, Germany, Spain, Finland, Greece, Ireland, The Netherlands, Norway, Poland and Sweden (Sermeus et al., 2011). The main aim of the RN4CAST study was to derive nurse forecasting models that consider how work environments' characteristics impact on nurse and patient outcomes. The RN4CAST study protocol was approved by either central ethical committees or local ethical committees, depending on each country's regulatory requirements. The study methods were compliant with the STrengthening the Reporting of OBservational studies in Epidemiology (STROBE) checklist (Appendix S1).

### 3.1 Data

Data were collected from registered nurses (referred to as “nurses” in this study for brevity) working in general hospitals within surgical, medical or mixed medical‐surgical wards between 2009–2010. Data collection differed between countries: a hospital field manager administered questionnaires to nurses; a hospital field manager held visits of the RN4CAST team to the sampled wards, who explained the study and distributed the questionnaires; nurses received the questionnaires via e‐mail; nurses received questionnaires by mail at their home address. Nurses were asked to return responses within 3 weeks. For the detailed RN4CAST study methodology, please see Sermeus et al., (2011).

### 3.2 Measurements

There were 118 questions in the RN4CAST survey, combined in different sections: “About your job,” which included questions around opportunities to engage in continuing education programmes and in discussions around patient care, and around continuity of care; “Quality and safety,” including statements relating to patient issues; “About your most recent shift at work in this hospital,” which measured length of shift and staffing levels; “About you,” recording demographic variables including age, gender and education level; and “Your job,” enquiring aspects including job title, play band and ward type.

Nurses' length of shift was captured by a question asking the number of hours worked on their most recent shift (i.e., the last shift they worked before filling in the questionnaire). To perform multilevel regression analysis, we categorised shift length into the following four groups: 8 hr or less; between 8.1–10 hr; between 10.1–11.9 hr; 12 hr or more. We created a variable to categorise day and night shifts and removed all subjects who provided invalid responses. Reponses to a question enquiring about overtime on the last shift were recorded as “yes” or “no,” and nurses indicated whether they were working full time or part‐time at the hospital. When nurses had indicated that a shift lasted 18 hr or more, we removed their responses from their dataset. Absolute numbers of these responses were low (*n* = 507, 1.3%). We chose this cut‐off because reported shifts longer than 18 hr are likely invalid answers based on nurses' total weekly hours. Four measures were drawn from the survey as study outcomes. Nurses were asked to what extent they agreed with the following statements: “there are active staff development or continuing education programmes for nurses in my current job”; “there is enough time and opportunity to discuss patient care problems with other nurses”; “there are patient care assignments that foster continuity of care (i.e., the same nurse cares for the patient from one day to the next).” These statements were rated on a 4‐item Likert scale, where 1 indicated “strongly disagree” and 4 “strongly agree.” For analysis, we grouped “somewhat agree” and “strongly agree” responses to reflect positive evaluations. The final question was “Important patient care information is often lost during shift changes” with responses “strongly disagree,” “disagree,” “neither,” “agree,” “strongly agree.” We grouped “strongly agree” and “agree” to reflect a negative evaluation (i.e., agree that important care is missed).

### 3.3 Data analysis

We first performed descriptive analyses, where outcomes were described using frequencies and percentages by shift length category. The association between shift length and nurses' reports of information and communication activities was explored using generalised linear mixed models, first by including country, hospital and ward as random effects.

We then added potential confounding variables to the models, including timing of last shift (i.e., day or night); presence of overtime; ward nurse staffing levels; hospital size; hospital technology status; hospital teaching status; full‐time/part‐time work; and nurses' age and gender. All models included country, hospital and ward as random effects.

To ensure no multicollinearity was present between the control variables, we computed the variance inflation factor (VIF) with VIF <5 indicating no multicollinearity (Dormann et al., 2013). All statistical analysis was undertaken with RStudio version 1.1.442 (R Development Core Team, 2018) and the lme4 package (Bates, Mächler, Bolker, & Walker, 2015). We adopted the Akaike information criterion (AIC) and the Bayesian information criterion (BIC) to evaluate relative model fit, prioritising models with lower values of AIC/BIC.

## 4 RESULTS

In total, 54,140 questionnaires were distributed and 33,659 (62%) nurses in 487 hospitals responded. After removing shift work invalid responses, our analytical sample totalled 31,627 nurses. Mean age of respondents was 38 years, and 93% were female. Detailed demographic description of the sample can be found elsewhere (Dall'Ora, Griffiths, Ball, Simon, & Aiken, 2015).

Half of the nurses in Europe worked shifts of 8 hr or less (*n* = 15,930, 50%). Overall, 9,963 nurses (31%) had worked between 8.1–10 hr on their last shift, while shifts of between 10.1 and less than 12 hr were reported only by 1,159 nurses (4%). Overall, 4,574 nurses (14%) reported that their last shift lasted 12 hr or more. The majority of nurses' worked day shifts (*n* = 24,627, 78%), and 8,606 nurses (27%) reported working beyond their contracted hours (i.e., overtime) on their last shift. Frequency of different shift length categories on the country level can be found elsewhere (Griffiths et al., 2014).

Fifty‐five per cent of nurses agreed that there were staff development or continuing education programmes offered within their work environments (*n* = 17,246), and 46% of the nurses agreed that there was enough time and opportunity to discuss patient care problems with other nurses (*n* = 14,481). In this sample, 21% agreed that important patient care information was lost during shift changes (*n* = 6,452); and 57% (*n* = 17,987) agreed that there were assignments that foster continuity of care in their job. Table 1 reports the nurses' responses by shift length category.

**Table 1:** Outcomes by shift length category

Outcomes	Shift length (hr) *n* (%)				
	≤8	8.1–10	10.1–11.9	≥12	All^a^
There are active staff development or continuing education programmes for nurses					
Agree	8,738 (55.4)	5,348 (54.2)	734 (64.3)	2,426 (53.5)	17,246 (55.1)
Total	15,748	9,858	1,141	4,528	31,275
There is enough time and opportunity to discuss patient care problems with other nurses					
Agree	7,619 (48.1)	4,324 (43.7)	567 (49.2)	1,971 (43.3)	14,481 (46.1)
Total	15,822	9,893	1,153	4,544	31,412
There are assignments that foster continuity of care					
Agree	9,366 (59.3)	5,694 (57.7)	685 (59.8)	2,242 (49.5)	17,987 (57.4)
Total	15,769	9,862	1,146	4,528	31,305
Important patient care information is lost during shift changes					
Agree	2,979 (18.9)	2,030 (20.5)	371 (32.2)	1,072 (23.7)	6,452 (20.6)
Total	15,766	9,873	1,151	4,512	31,302

a Total number of responses does not equal to 31,627 due to invalid responses.

Long shifts were associated with decreases in the odds of reporting beneficial outcomes (Table 2). Working shifts of 12 hr or more was associated with a decrease in the odds of nurses agreeing that there were enough active staff development or continuing education programmes, when compared to working 8 hr or shorter (adjusted odds ratio [aOR]: 0.69; 95% confidence interval [CI]: 0.59–0.80). The odds of nurses agreeing to have enough time and opportunity to discuss patient care problems with colleagues were reduced for nurses working shifts of 8 hr or more compared to nurses working shifts of 8 hr or less. For nurses working shifts of 12 hr or more, the odds of reporting being able to discuss patient care were decreased by 24%, in comparison with nurses working 8 hr or less (aOR: 0.76; 95% CI: 0.66–0.87).

**Table 2:** Outputs of generalised linear mixed models measuring the association between shift characteristics and outcomes of information and communication flow

Outcome	Random effects only		Fully adjusted^a^	
	Odds ratio	95% CI	Odds ratio	95% CI
Active staff development or continuing education programmes for nurses				
≤8 hr shift (reference category)				
8.1–10 hr shift	0.97	0.91–1.03	1.02	0.94–1.11
10.1–11.9 hr shift	0.86	0.73–1.01	0.86	0.71–1.04
≥12 hr shift	0.65*	0.57–0.74	0.69*	0.59–0.80
Enough time and opportunity to discuss patient care problems with other nurses				
≤8 hr shift (reference category)				
8.1–10 hr shift	0.84*	0.80–0.89	0.92*	0.85–0.99
10.1–11.9 hr shift	0.82*	0.71–0.94	0.79*	0.66–0.93
≥12 hr shift	0.76*	0.67–0.86	0.76*	0.66–0.87
Assignments that foster continuity of care				
≤8 hr shift (reference category)				
8.1–10 hr shift	0.87*	0.82–0.92	0.96	0.89–1.04
10.1–11.9 hr shift	0.93	0.81–1.08	0.96	0.80–1.14
≥12 hr shift	0.94	0.83–1.07	0.97	0.83–1.12
Important patient care information is lost during shift changes				
≤8 hr shift (reference category)				
8.1–10 hr shift	1.25*	1.16–1.34	1.09	0.99–1.19
10.1–11.9 hr shift	1.15	0.98–1.35	1.01	0.83–1.21
≥12 hr shift	1.28*	1.11–1.47	1.11	0.95–1.30

a Controlling for type of shift (day vs. night); overtime; technology status; hospital size; ward type (medical vs. surgical); teaching status; nurse staffing levels; nurse age; nurse gender; working full‐time/part‐time. Random effects: country; hospital; ward

* Significant at *p* < 0.05.

Working shifts of 12 hr or more was not associated with increases in the odds of nurses reporting assignments that foster continuity of care (aOR: 0.97; 95% CI: 0.83–1.12), when compared to working 8 hr or less. Working shifts of 12 hr or more was associated with nurses' reports of important patient care information being lost during shift handovers in comparison with working shifts of 8 hr or less (odds ratio [OR]: 1.28; 95% CI: 1.11–1.47), although the association was attenuated when controlling for other shift variables, nurses' demographics and ward/hospital characteristics (aOR: 1.11; 95% CI: 0.95–1.30). All models' variance inflation factors confirmed that multicollinearity was not present at a problematic level.

## 5 DISCUSSION

This study is one of the first to examine the association between long shifts and aspects of nursing work, including ability to participate in continuing education activity and discuss patient care; and aspects of quality of care, including continuity of care and information loss during handover in Europe using a multi‐country multilevel design. Shifts of 12 hr or more are common in some European countries, especially in Poland, Ireland and the United Kingdom (Griffiths et al., 2014), where they are increasingly being implemented based on assumptions of cost savings and improved quality of care (NHS Evidence, 2010). This study challenged this assumption. Drawing on a large and diverse European sample of 31,627 nurses, and controlling for a number of potential confounders, we found that nurses working long shifts were less likely to report having the opportunity to participate in continuing educational opportunities and to discuss patient care. Although nurses who worked long shifts were more likely to report that important patient information was being lost during handovers and not having assignments that fostered continuity of care, these associations were not statistically significant.

Our findings confirmed those of small scale qualitative studies, highlighting that working on long shift patterns and, therefore, losing the long overlap between shifts, leads to fewer opportunities to engage in educational activities and in discussions around patient care (McGettrick & O'Neill, 2006). Contrary to reports that long shifts foster continuity of care and reduce information loss (Haller et al., 2018; NHS Evidence, 2010; Wootten, 2000), our study found no significant associations between working shifts of 12 hr or more and continuity of care and information loss.

There is evidence that some nurses perceive long shifts as beneficial (Stone et al., 2006). The main reasons for nurses preferring long shifts were the ability to compress the working week into 3 days rather than five, thus benefitting from more days off; better work–life balance; and reduced travel costs (Harris, Sims, Parr, & Davies, 2015). Our results suggest that nurses may choose to work longer but fewer shifts, but this appears to be at the expense of continuing education programmes, and the ability of engaging in conversations around patient care. Nurses have indicated that participating in continuous professional development is pivotal to their job satisfaction and the quality of care they provide (Price & Reichert, 2017), outcomes that have been reported to be affected by long shifts (Dall'Ora et al., 2015; Griffiths et al., 2014).

Our study shows that shifts of 12 hr or more were associated with missed opportunities to discuss patient care amongst nurses. This mirrors evidence that nurses' long shifts are associated with increased missed care (Ball et al., 2017; Griffiths et al., 2014). When nurses are experiencing competing demands during a shift, they may choose to prioritise clinical activities, including patient surveillance and treatments and procedures, at the expense of planning and discussing patient care (Griffiths et al., 2018).

If nurses working shifts of 12 hr or more do not have sufficient time, energy and opportunity to participate in educational activities and to discuss patient care, the quality of care they provide may be lower. A recent study found that shifts of 12 hr or more are not associated with reduced staffing hours per patient day and staffing costs (Griffiths, Dall'Ora, Sinden, & Jones, 2019), and there is evidence that shifts of 12 hr or more are associated with increased sickness absence for nurses (Dall'Ora, Ball, et al., 2019). The evidence that long shifts do not lead to a decrease in resource use, combined with our study's findings, suggests that the hypothesised beneficial effect of long shifts is not achieved.

### 5.1 Limitations

This study had some limitations. First, it relied on cross‐sectional data, so no assumptions that the relationship between long shifts and outcomes is causal should be made. Second, the data we drew on were nurse‐reported, which may have led to nurses interpreting the outcomes subjectively. Shift length was investigated for the nurses' most recent shift only, which may not be representative of all nurses' shifts. Furthermore, the RN4CAST study did not aim to study shift work in depth, therefore shift characteristics that should be considered were not included in the survey. Lastly, the data were collected between nine and ten years ago, and shift patterns may have changed across Europe indicating that our data may not be current; however, we are not aware of any substantial changes in any healthcare systems, with the exception of England where shifts of 12 hr or longer are more common than they were ten years ago (Royal College of Nursing, 2017).

## 6 CONCLUSIONS

The present study found no evidence for beneficial effects of shifts of 12 hr or more in terms of continuity of care or reduced patient information being lost during handover. Contrastingly, shifts of 12 hr or more shifts are associated with reduced opportunities to discuss care and participate in continuing education activities. As well as negatively impacting quality of care, there may be more insidious effects of shifts of 12 hr or more, especially when considering the long term and cumulative impact of not being able to participate in educational activities and discuss patient care.

## 7 RELEVANCE TO CLINICAL PRACTICE

This study's findings have implication for nurses: shifts of 12 hr or more are likely to jeopardise a number of important activities for nurses. When such findings are considered in light of a large body of literature highlighting the negative effect long shifts have on nurse and patient outcomes, nurses should consider whether the benefits of working shifts of 12 hr or more outweigh the risks to their professional practice and their patients' safety.

Nurse managers make daily decisions on how to best staff hospital wards, and shift patterns are one of the aspects they are required to consider, to ensure that the care provided is of high quality and safe, and that staff wellbeing is not compromised. Our findings should encourage nurse managers to question the implementation of long shifts and to explore alternative options and solutions with nurses. If shifts of 12 hr or more cannot be avoided, nurse managers should ensure that protections are in place, including nurses' ability to take breaks; the provision of appropriate staffing levels; no more than three consecutive long shifts are scheduled (Thomson et al., 2017). Nurse managers should also explore how to compensate for reduced opportunities to undertake continuous education within shifts. Healthcare is changing rapidly, and nurses' knowledge cannot be simply acquired at the bedside. There is ample evidence that when nurses have access to education programmes at work, patient outcomes are improved (Gallagher, 2007). Therefore, if nurses are not able to access education programmes and keep up to date with best evidence‐based practice, patient may not receive safe and effective care.

## CONFLICT OF INTEREST

The authors declare that they have no conflict of interest.

## Appendix 1

### The RN4CAST consortium

Walter Sermeus (Director), Koen Van den Heede, Luk Bruyneel, Emmanuel Lesaffre (Belgium, Catholic University Leuven); Linda Aiken (Co‐Director), Herbert Smith, Douglas Sloane (USA, University of Pennsylvania); Anne Marie Rafferty (UK, King's College London), Simon Jones (UK, University of Surrey) Jane Ball, Peter Griffiths (UK, University of Southampton and Sweden, Karolinska Institutet); Juha Kinnunen, Anneli Ensio, Virpi Jylhä (Finland, University of Eastern Finland); Reinhard Busse, Britta Zander, Miriam Blümel (Germany, Berlin University of Technology); John Mantas, Marianna Diomidous (Greece, University of Athens); Anne Scott, Anne Matthews, Anthony Staines (Ireland, Dublin City University); Ingeborg Strømseng Sjetne (Norwegian Knowledge Centre for the Health Services) Inger Margrethe Holter (Norwegian Nurses Organization); Tomasz Brzostek, Maria Kózka, Piotr Brzyski (Poland, Jagiellonian University Collegium Medicum); Teresa Moreno‐Casbas, Carmen Fuentelsaz‐Gallego, Esther Gonzalez‐María, Teresa Gomez‐Garcia (Spain, Institute of Health Carlos III); Carol Tishelman, Rikard Lindqvist, Lisa Smeds‐Alenius (Sweden, Karolinska Institutet); Sabina De Geest, Maria Schubert, René Schwendimann; Dietmar Ausserhofer (Switzerland, Basel University); Theo van Achterberg, Maud Heinen, (Netherlands, Radboud University Nijmegen Medical Centre), Lisette Schoonhoven (Netherlands, Utrecht University).
